# Diode Laser Stapedotomy: Audiological Results and Clinical Safety

**DOI:** 10.3390/audiolres16010022

**Published:** 2026-02-02

**Authors:** Daniela Parrino, Guglielmo Romano, Graziano Pavan, Paolo Castelnuovo, Maurizio Bignami

**Affiliations:** 1 Department of Otorhinolaryngology Head and Neck Surgery, ASST Sette Laghi, Ospedale di Circolo e Fondazione Macchi, 21100 Varese, Italy; guglielmo.romano@asst-settelaghi.it (G.R.); graziano.pavan@asst-settelaghi.it (G.P.); paolo.castelnuovo@uninsubria.it (P.C.); maurizio.bignami@uninsubria.it (M.B.); 2 Head and Neck Surgery & Forensic Dissection Research Center (HNS&FDRc), Department of Biotechnology and Life Sciences, University of Insubria, 21100 Varese, Italy

**Keywords:** stapedotomy, stapes surgery, otosclerosis, diode laser, audiology

## Abstract

*Background and objectives:* Stapedotomy is the surgical treatment for otosclerosis, with excellent results in terms of hearing recovery. Various laser systems have proven to be an interesting alternative to the conventional technique, allowing for a more precise footplate fenestration with apparently less trauma to the inner ear. The diode laser, more recently introduced, seems to offer more controlled tissue interaction, potentially reducing thermal damage to surrounding structures. However, the literature remains limited. The aim of the present study was to evaluate the functional outcomes and clinical safety of diode laser stapedotomy by comparing the observed results with those previously reported. *Materials and methods:* A retrospective analysis of 105 patients who underwent diode laser stapedotomy was conducted. The audiological data and the complications were analyzed and compared with a cohort of patients who underwent stapedotomy performed using the conventional technique. *Results:* In patients who underwent diode laser stapedotomy, the postoperative air–bone gap (ABG) improved significantly at all frequencies. Hearing outcomes were excellent (ABG ≤ 10 dB) in 60.9% of cases, good (ABG ≤ 20 dB) in 89.5%, and poor (ABG > 20 dB) in 10.5% of patients. Intraoperative complications occurred in seven patients (6.7%), including two cases (1.9%) of footplate damage. Postoperatively, 13 cases of vertigo (12.4%), three cases of tinnitus (2.8%), and one case of sensorineural hearing loss (0.9%) were reported. *Conclusions:* Diode laser stapedotomy is an effective and safe procedure, providing excellent audiological outcomes without increasing the risk of surgical complications. The possibility of thermal damage to the inner ear must be considered, and appropriate laser parameters should be used to minimize these risks.

## 1. Introduction

Otosclerosis is a bony labyrinth disease caused by structural changes in the otic capsule, which leads to stapes fixation and results in progressive conductive hearing loss (CHL) [1]. Stapedotomy is the treatment of choice for CHL caused by otosclerosis, with the aim of restoring the ossicular chain movements using a prosthesis, after removing the stapes superstructure and opening the perilymphatic space of the vestibule by stapes footplate perforation [2]. Despite being a safe procedure, stapedotomy is not risk-free and may cause permanent sensorineural hearing loss (SNHL) in a small percentage of patients [3]. Since the first description of the oval-window fenestration by Shea in 1956 [4] to treat otosclerosis, stapes surgery has been revolutionized to be an increasingly less-invasive procedure. Small fenestration techniques (stapedotomy), with different instruments (i.e., micro-perforators, microdrills, and more recently, lasers), were developed to reduce the significant risk of inner ear damage associated with the original removal of the entire stapes footplate (total stapedectomy) or one-third of the footplate (partial stapedectomy).

In particular, the laser was first introduced in otology by Sataloff in 1967 during experimental studies on stapedotomy [5]. Since then, clinical studies using different types of lasers in otosclerosis—such as argon, carbon dioxide (CO_2_), potassium-titanyl-phosphate (KTP), and erbium—have been reported, showing equivalent or improved audiological results in comparison to the standard technique [6,7,8,9,10]. The main advantage of laser is its precision, with a low risk of footplate mobilization and potential inner ear damage [11]. Besides that, other potentially deleterious effects such as thermal damage, heating of the perilymph and of neighboring structures such as the facial nerve should be kept in mind [11]. Regarding the diode laser in stapes surgery, it was introduced in the 2000s [12,13], later than the other abovementioned lasers, with the expectation of becoming an efficient instrument for the procedure, given its characteristic of being contact/near-contact, with a very good hemostatic effect and high precision for incisions [12]. This allows for more controlled tissue interaction, potentially reducing thermal damage to surrounding structures [10]. To date, there are few studies available in the literature reporting diode laser use in stapes surgery.

The aim of the present investigation was to evaluate the functional results and clinical safety outcomes of the diode laser in stapedotomy, comparing the findings with those already published. The results were additionally compared with a series treated by the conventional technique.

## 2. Materials and Methods

### 2.1. Patients

The study was conducted in accordance with the principles of the Helsinki Declaration. Data were examined in compliance with Italian privacy and sensitive data laws, and with the in-house rules of the Department of Otorhinolaryngology Head and Neck Surgery, ASST Sette Laghi, Varese, Italy.

A retrospective analysis of the medical charts of all adult patients (over 18 years of age) who underwent stapes surgery for otosclerosis at our institution between 1 May 2009 and 31 December 2023 was performed. Diagnosis of otosclerosis was made according to clinical examination and audiological criteria. Otoscopy was normal, tympanometry was type As, and the stapedial reflex was absent in all cases. Indications for surgical management included conductive or mixed hearing loss with a >25 dB pure-tone air–bone gap (PTA-ABG).

Patients who underwent revision surgery were excluded. Patients in whom stapes fixation was not confirmed during the surgical procedure, those with tympanosclerosis, or those with other ossicular chain abnormalities were also excluded. Additional exclusion criteria were concomitant chronic ear infection, tympanic membrane perforation or retraction pocket, hematological diseases, active cancer, or other diseases that could affect the data of the study. Patients with incomplete information were finally ruled out.

Demographic and clinical data, relevant information from the surgical description, intraoperative and postoperative complications, and audiological tests performed before and after surgery were recorded for all patients.

### 2.2. Diode Laser Characteristics

A wavelength 940 nm diode laser delivered via a fiber of 0.4-millimeter-diameter tip coupled to a hand piece (Dornier D Fibertom; Dornier MedTech GmbH, Wessling, Germany) was used. The laser tip was systematically burned on wooden tongue depressor before use. The energy was delivered with direct contact to the targeted tissue—stapedial tendon, stapes posterior crura and stapes footplate—to vaporize them. A single pulse, powered at 40 W and lasting 60 msec, was applied.

### 2.3. Surgical Technique

All surgeries were performed by two senior surgeons. The procedure was conducted under local anesthesia, with patients sedated, by a transcanal microscopic approach. In selected cases, general anesthesia was preferred, or an endaural approach was chosen for particularly narrow external auditory canals (EACs). A skin incision of the EAC was made, and the tympanomeatal flap elevated until reaching the tympanic cavity. The chorda tympani nerve was located, and the scutum curetted to expose the structures on the stapes footplate, the horizontal segment of the facial nerve, and the stapes tendon. The ossicular chain motility was evaluated, and otosclerosis confirmed by fixation of the stapes with mobile malleus and incus. The distance between the long process of the incus and the stapes footplate was measured, and the length and type of prosthesis were defined. The incudo-stapedial joint was disarticulated, the tendon of the stapes muscle and the posterior crura were vaporized, and then the supra-structure of the stapes was removed by fracturing the anterior crura using an angled hook. Therefore, the entire footplate was exposed, and a single diode laser pulse (40 W, 60 msec) was shot to fenestrate the footplate. If the first pulse was not sufficient, second or third pulses were delivered to complete footplate fenestration. An appropriately sized piston with a 0.4-millimeter-diameter shaft was implanted and clipped to the long process of the incus. The activity of the reconstructed ossicular chains was tested. The tympanic flap was then reverted, and the EAC filled with gelfoam.

The technique described above is the classic stapedotomy. Surgical steps can be interchanged and the prosthesis positioned before removing the stapes supra-structure (reversal technique). In some cases, we performed a so-called “modified classic technique” in which incudo-stapedial joint disarticulation and vaporization of the stapes muscle tendon and the posterior crura were first performed, while the footplate fenestration was performed before fracturing the anterior crura to remove the entire stapes supra-structure; then, the prosthesis was finally positioned.

Regarding the cases performed with the conventional technique, the stapes tendon was cut by microscissors, and the posterior crura were drilled out using a microdrill, as well as the fenestration of the stapes footplate.

### 2.4. Audiological Results

All patients underwent pure-tone audiometry preoperatively and 6 months postoperatively. Pure-tone average (PTA) thresholds were calculated at 0.5, 1, 2, and 4 kHz. The following parameters were evaluated: (i) Preoperative and postoperative PTA air conduction (AC). (ii) Preoperative and postoperative PTA bone conduction (BC). (iii) Preoperative and postoperative mean air–bone gap (ABG). (iv) ABG closure. Surgical success was categorized according to the guidelines for reporting results in stapes surgery of the American Academy of Otolaryngology–Head and Neck Surgery (AAOHNS 1995) [14]: a residual postoperative ABG ≤ 10 dB was defined as an excellent result; a residual postoperative ABG ≤ 20 dB and >20 dB were defined as good and poor results, respectively. (v) Mean BC threshold variation between preoperative and postoperative audiometry; sensorineural damage was considered a drop of BC-PTA threshold ≥ 15 dB in the postoperative audiometry. (vi) BC threshold variation between preoperative and postoperative pure-tone audiometry at 4 kHz.

### 2.5. Clinical Safety

The clinical safety outcome was established as the assessment of sensorineural damage (decrease of BC-PTA ≥ 15 dB in the postoperative audiometry). Any postoperative changes in the sensorineural (BC) hearing threshold at high frequency, 4 kHz, was also specifically evaluated.

Secondary outcome measures were intraoperative and postoperative complications.

### 2.6. Statistical Analysis

Statistical analyses were performed using R Statistical Software (ver. 3.6.2, Foundation for Statistical Computing). Quantitative variables are described as mean ± standard deviation and median, whereas categorical variables are reported as numbers and percentages. Because our data did not follow a normal distribution, the mean preoperative and postoperative values were compared using the non-parametric Mann–Whitney test. The comparison of the main outcomes according to the subgroups was analyzed by a simple linear regression model. For categorical variables, Fisher’s exact test was used. A *p*-value < 0.05 was considered indicative of statistical significance.

## 3. Results

### 3.1. Patients

A total of 219 eligible patients were selected. After applying exclusion criteria, 78 cases were excluded, and 141 patients were considered for the analysis. In the initial period, when laser was not available, 36 surgeries (25.5%) were performed with the conventional technique by microdrill; the remaining 105 surgeries were performed by diode laser, which is the focus of our analysis.

Table 1 summarizes demographic and surgical characteristics of all patients.

### 3.2. Audiological Results of Diode Laser Stapedotomy Patients

The audiological results of patients who underwent diode laser stapedotomy are shown in Table 2. On preoperative pure-tone audiometry, mean ± SD (median, IQR) AC-PTA and mean ± SD (median, IQR) BC-PTA were 58.02 ± 12.58 (56.25, 12.5) decibels (dB) and 26.45 ± 10.54 (25.0, 12.5) dB, respectively. Preoperative ABG was 31.57 ± 2.03 (31.25, 11.25) dB. Postoperatively, the mean ± SD (median, IQR) AC-PTA was 35.45 ± 13.54 (32.5, 21.25) dB, the mean ± SD (median, IQR) BC-PTA was 23.64 ± 10.86 (20, 16.25) dB, and the ABG was 11.81 ± 2.68 (8.75, 6.25) dB.

**Figure 1:**
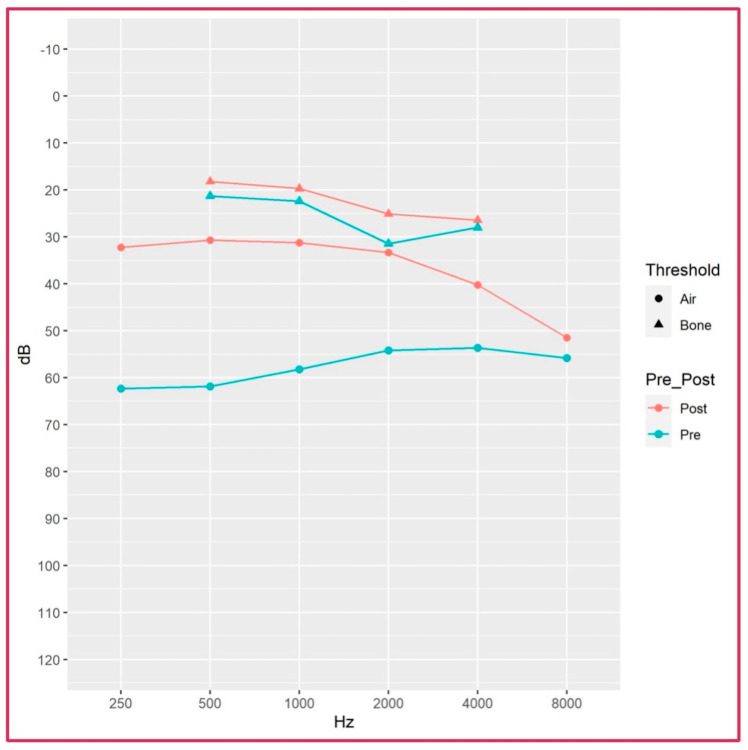
Representation of preoperative and postoperative audiometric data of average values of patients who underwent diode laser stapedotomy.

The statistical comparison between preoperative and postoperative audiological values is reported in Table 3. The analysis shows a statistically significant hearing improvement for all AC frequencies with an average gain of 22.7 dB (range 12.7–28 dB), as represented in Figure 1. The BC threshold also improved, despite lacking statistical significance. The postoperative ABG was also improved for all frequencies with statistical significance (Table 3).

**Figure 2:**
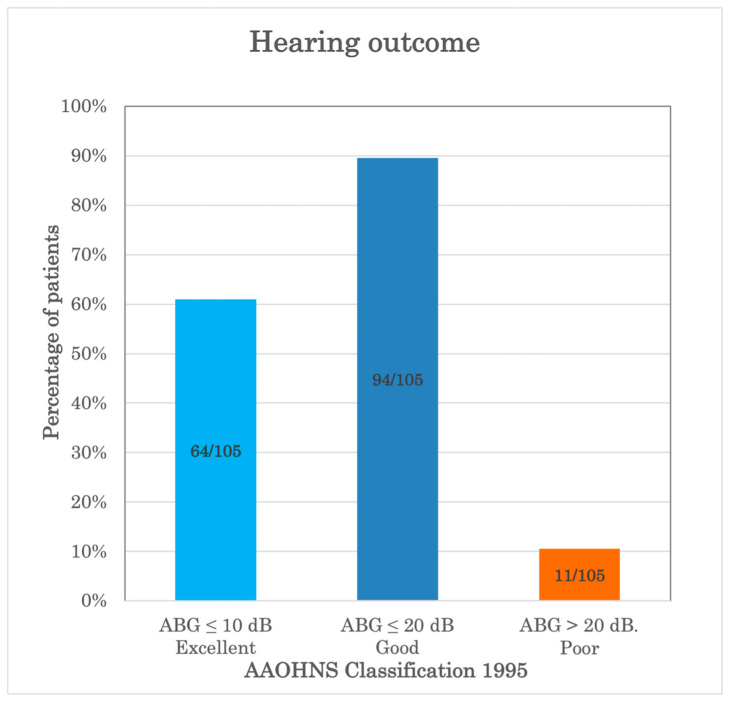
Postoperative air–bone gap hearing results of diode laser stapedotomy, according to the classification suggested by the American Academy of Otolaryngology and Head and Neck Surgery (AAOHNS 1995) [14]. ABG: air–bone gap.

Figure 2 categorizes the postoperative ABG results according to the recommendations of the AAOHNS [14]. The hearing outcome was excellent in 64 out of 105 patients (60.9%), good in 94 out of 105 patients (89.5%), and poor in the remaining 11 patients (10.5%).

### 3.3. Subgroups Analysis of Diode Laser Stapedotomy Patients

When patients were analyzed according to gender (male and female), age (younger or older than 50 years old), or surgical steps (classic, reversal, or modified classic), a statistically significant improvement in the AC threshold and ABG, along with an absence of worsening in the BC threshold were observed in each subgroup. Statistical analysis ruled out any significant differences in terms of preoperative and postoperative auditory outcomes between the subgroups (Table 4). Only patients aged ≥50 years old showed a minor improvement in the postoperative ABG at 2 kHz in comparison to the younger patients, with statistical significance (*p* = 0.018).

### 3.4. Complications of Diode Laser Stapedotomy Patients

Seven patients out of 105 (6.7%) had intraoperative complications. One patient (0.9%) had a gusher. Two patients (1.9%) had footplate damage (one floating footplate and one footplate fracture), which occurred during the removal of the stapes supra-structure by using the angled hook. One patient (0.9%) had a strong and long-lasting episode of vertigo, and two patients showed a tympanic membrane laceration, which was immediately repaired without subsequent evidence of postoperative residual perforation.

Postoperatively, 13/105 patients (12.4%) and 3/105 patients (2.8%) complained of vertigo and tinnitus, respectively, which completely resolved in all cases. One patient (0.9%) showed sensorineural hearing loss (SNHL), with a decrease of BC-PTA ≥ 15 dB in the postoperative audiometry compared to preoperatively.

The evaluation of postoperative BC 4 kHz threshold showed an impairment of ≥15 dB in six patients (5.7%), and in two of those, the impairment was ≥20 dB (1.9%).

Analyzing the BC thresholds of the four patients who experienced intraoperative complications risky for inner ear damage (floating footplate, fractured footplate, gusher, and vertigo), none had postoperative SNHL.

### 3.5. Conventional Technique Versus Laser Diode Technique

Table 3 shows the audiological data of patients treated by the conventional microdrill technique. There was a statistically significant improvement in postoperative hearing in comparison with preoperative values for almost all frequencies.

Both groups of patients presented with benefit in the audiological postoperative results when compared to the preoperative period. The comparative statistical analysis between the diode laser group and the conventional technique group ruled out any statistically significant difference in audiological outcome (linear regression model, all *p*-values > 0.05).

Regarding the complications that occurred in the 36 conventional technique cases, one patient (2.7%) had intraoperative vertigo, one (2.7%) postoperative transient vertigo, and one (2.7%) postoperative SNHL. Fisher’s exact test showed no significant difference in the incidence of complications between the two techniques (*p*-values > 0.05).

Considering the numerical variability in the two samples of our series, evidence of differences may not be observed.

## 4. Discussion

Since the first description of the oval-window fenestration by John Shea in 1956 [4] to treat otosclerosis, stapes surgery has been revolutionized as an increasingly less invasive procedure. Small fenestration techniques (stapedotomy) were developed to reduce the significant risk of inner ear damage associated with the original removal of the entire stapes footplate (total stapedectomy) or one-third of the footplate (partial stapedectomy). In particular, the laser was first applied in stapedotomy by Sataloff in 1967 [5] during experimental studies. Palva in 1979 [15] and Perkins in 1980 [6] were then the first surgeons to use the argon laser in clinical practice for perforation of the footplate and, during the next year, the technique increased in popularity due to its precision in creating the stapes fenestration, with the advantage of reducing the mechanical trauma to the inner ear due to footplate mobilization [5,6]. Subsequently, clinical studies using different types of lasers—such as argon, carbon dioxide (CO_2_), potassium-titanyl-phosphate (KTP), and erbium—in otosclerosis surgeries have been reported and have shown comparable audiological outcomes to the conventional technique, with acceptable rates of complications [8,9,10,11,16,17].

The diode laser was introduced later in otologic surgery, and clinical reports attesting to its efficacy and clinical safety are still few in the literature, as shown in Table 5. Nguyen et al. [18] in 2008 conducted a retrospective analysis of 112 patients who underwent operations for otosclerosis with diode laser and 141 with the conventional technique, observing comparable audiological outcomes between the two groups and a decreased incidence of footplate fracture in the laser group (3.6%, 4/112) in comparison to the microdrill group (21.3%, 30/141). This evidence had already been reported with other laser types [19,20].

Although many authors agree that lasers in general have improved the surgical precision in stapes surgery, decreasing mechanical and vibratory trauma, the choice of laser type is also significant. Kalmaski et al. [10] reviewed various laser types used in stapedotomy and found that diode lasers, with a specific wavelength of 980 nm, allow for more precision and more controlled tissue interaction, potentially reducing the thermal and acoustic effects to the surrounding structures; this may provide specific benefits over argon, CO_2_, and KTP lasers. Additionally, the diode laser fiber delivery system with an ergonomic hand piece provides better accuracy, and, with respect to other lasers, is relatively inexpensive [10,18].

In a double-blinded randomized clinical trial, Parida et al. [21] directly compared diode laser stapedotomy with conventional stapedotomy. Their results indicated that patients who underwent diode laser stapedotomy experienced similar or better audiological outcomes, reinforcing the notion that diode laser can be a viable alternative to traditional methods. This is particularly relevant given the aforementioned increasing demand for minimally invasive surgical options that can enhance patient recovery and satisfaction.

The concept of the “One Shot” diode laser stapedotomy, as described by Poletti et al. [22], further illustrates the innovation in this field. The authors retrospectively analyzed 179 primary cases of otosclerosis patients who underwent diode laser stapedotomy. Diode lasers with 940 nm and 980 nm wavelengths set to 40 W for a short exposure time of 60 msec were applied. The study showed good audiological results and excluded the possibility that diode lasers with the wavelengths and parameters used could be dangerous for the inner ear, confirming that this stapedotomy technique is safe and effective.

An advantage of the diode laser technique was further discussed by other authors comparing the conventional and laser techniques [23,24]. Although there were no significant differences in the audiometric results, patients who underwent diode laser stapedotomy tended to show a lower frequency of complications, such as sensorineural damage and postoperative vertigo, and often experienced shorter recovery times [23,24]. Additionally, Plodpai in 2022 [25] described 56 exclusive endoscopic diode laser stapedotomies, assessing that it is an effective and safe minimally invasive technique that provides satisfactory hearing outcomes with low postoperative complications. The fiberoptic handheld delivery system is suitable for an endoscopic approach; it is easy to utilize and is a cost-effective device [25]. More recently, another study analyzed the differences between microdrill and diode laser instruments in endoscopic stapedotomy for otosclerosis, showing the absence of postoperative complications and an equivalent improvement in hearing level in the two groups, although the surgical time was shorter in the microdrill group [26]. Thus, they supported the use of a microdrill over a diode laser in stapes surgery. In our opinion, the surgeon’s experience with one technique rather than another may have influenced the surgical time in this case.

**Table 5:** Comparison of diode laser reports in the literature.

Authors	Stapedotomy	N° Cases	ABG ≤10 dB	ABG ≤20 dB	SNHL ≥15 dB	SNHL ≥20 dB	Footplate Damage
Nguyen et al. 2008 [18]	Diode/Conventional	112/141	72/85%	93/95%	-	1.8/2.8%	3.6/21.3%
Poletti et al. 2015 [22]	Diode	179	89%	97%	-	-	-
Parida et al. 2016 [21]	Diode/Conventional	30/30	-	86.7/83.3%	-	-	-
Plodpai 2022 [25]	Diode		91%	96.4%	-	-	3.5%
Wang et al. 2024 [26]	Diode/Conventional	62/69	30.4/40.3%	-	-	-	-
Ordóñez Ordóñez et al. 2024 [24]	Diode/Conventional	56/97	91.1/82.5%	100/97.9%	3.9/16.5%	-	-
Present series	Diode/Conventional	105/36	60.9/47.2%	89.5/97.2%	0.9%/2.7%	-	1.9/0%

ABG: air–bone gap; SNHL: sensorineural hearing loss.

A multicenter retrospective study on 615 patients was recently published that evaluated long-term audiological outcomes and predictive factors of success in stapes surgery across three surgical techniques: diode laser stapedotomy, microdrill stapedotomy, and combined KTP laser–microdrill stapedotomy [27]. While all the examined techniques showed excellent audiological results, postoperative ABG was significantly better with laser-based procedures compared to the microdrill alone [27].

Table 5 summarizes the diode laser stapedotomies series reported in the literature, and our results are consistent with those, even though the percentage of excellent results is slightly lower. In our study, postoperative ABG closure ≤ 10 dB and ≤20 dB was observed in 60.9% and 89.5% of patients treated by diode laser, respectively. The conventional technique has shown similar audiological results (ABG closure ≤ 10 dB in 47.2% and ≤20 dB in 97.2%), without evidence of statistically significant differences (*p* > 0.05). In the subgroup analysis, we observed that patients aged ≥ 50 years old showed a lower postoperative ABG recovery at 2 kHz compared to younger patients, with statistical significance (*p* = 0.018); this could be related to a possibly higher presence of initial sensorineural involvement in older individuals, who generally benefit from the surgical intervention.

Complications were rare and comparable with those of other laser series [18,19,20,25]. The incidence of postoperative SNHL was 0.9%, while footplate damage was observed in two cases (1.9%; one fracture and one floating footplate). It is worthy of note that the footplate damage occurred during the removal of the stapes supra-structure by using the angled hook, and in the diode laser series reported by Nguyen et al. [18], the footplate fractures occurred during fenestration calibration by manual instrument. This confirms that one advantage of laser stapedotomies is the reduction in the incidence of footplate fractures when compared to the conventional technique. Possible thermal damage caused by the laser to the inner ear remains a concern. Although in our cases we had a very low incidence of SNHL, when we considered specifically the frequency 4 kHz, the postoperative BC threshold decreased in 5.7% of patients, suggesting a negative thermal effect of the laser on that frequency. The potential of high frequencies worsening stapes surgery is still debated, and up to 6.5% is described [28,29]. However, Poletti et al. [22] observed a statistically significant improvement in the PTA-BC and in hearing loss at 8 kHz, reporting that this could be related to the absence of acoustic and thermal side effects of the laser setting used. Similar to Poletti et al. [22], we used the diode laser with a high power (40 W) and a low exposure time (60 msec). The high energy over a short exposure time allows the use of the laser alone to realize the fenestration and limits the thermal conduction to the perilymph and the inner ear [30]. The facial nerve is also at risk of laser thermal damage; four cases of delayed facial palsy after diode laser stapedotomy have been described, with complete recovery within one month [24].

Postoperative transient vertigo was observed in 12.4% of patients, similar to other diode laser series [18,22,25]. We reported tinnitus in 2.8%, in contrast to the 14.2% rate shown in another series [25]. Our rate of postoperative tinnitus may be underestimated in relation to its effective incidence, and this could represent an issue of transient and mild thermal damage that is laser-related; further research in this field is needed. A recent cadaveric study [31] showed that diode laser stapedotomy can generate substantial intracochlear-pressure transients, particularly at higher energy settings, potentially contributing to cochlear injury. Shorter pulse durations and lower power settings produced more predictable and linear pressure responses [31]. These findings highlight the importance of optimizing laser parameters to reduce potentially harmful intracochlear-pressure fluctuations during stapedotomy, and further studies are needed to determine the clinical impact of these pressure changes [31].

The present investigation evaluated the functional and clinical safety outcomes of diode lasers in stapedotomy, adding a contribution to the currently scarce literature. The results were additionally compared with our previous patients treated by the conventional technique. It should be emphasized that the present study was not designed to demonstrate superior audiological outcomes of diode laser-assisted stapedotomy compared with the conventional technique, as both approaches provide comparable hearing results when performed according to established surgical principles. Rather, the primary objective was to assess the safety of diode laser use during stapedotomy, with particular attention to its potential role in reducing intraoperative complications, such as stapedial footplate trauma. Our findings may provide clinically relevant insights for otologic surgeons by further supporting the safe use of diode laser technology in stapes surgery.

From an economic perspective, it should be acknowledged that stapedotomy using a diode laser may involve higher procedural costs than the conventional microdrill technique, mainly due to equipment acquisition, maintenance, disposable fibers, and safety requirements. In contrast, the microdrill technique typically relies on widely available, largely amortized instruments with relatively low incremental costs per procedure. No significant differences in complication rates were observed between the two techniques, so potential cost savings from reduced postoperative morbidity could not be demonstrated. While the laser may offer technical advantages in selected cases, its higher costs do not appear clearly offset by measurable clinical benefits. However, in comparison to other laser systems, such as CO_2_, diode lasers are generally less expensive to acquire and maintain, making them suitable even for low-volume otologic surgery centers [25]. Unfortunately, robust and standardized economic data directly comparing the costs of laser-assisted procedures are currently lacking in the literature. Further prospective studies incorporating detailed economic evaluations are warranted to better define the cost-effectiveness of diode laser-assisted stapedotomy.

The main limitations of the study concern the retrospective setting and the limited number of patients. The retrospective data collection entails the loss of numerous patients at follow-up and the risk of missing information. Particularly, vocal audiometry data were not available for most patients; thus, they were not considered in the analysis. Another limitation is the absence of standardized guidelines for patient referral for stapedotomy, resulting in variability in preoperative hearing loss criteria and, consequently, in postoperative hearing outcomes. Among the strengths, there is the homogeneity in the diode laser setting to perform stapes fenestration.

## 5. Conclusions

Diode laser stapedotomy is an effective and safe procedure to treat otosclerosis, providing excellent audiological outcomes and potential procedural benefits, including enhanced surgical precision. However, it does not confer definitive advantages over the conventional microdrill technique, and the possibility of thermal damage to the inner ear should be considered; appropriate wavelength and delivery energy parameters should be used to minimize that. Its cost-effectiveness should also be considered.

## Figures and Tables

**Table 1:** Demographic and surgical characteristics of patients.

Characteristic	All Cases (N = 141)	Laser Cases (N = 105)	Microdrill Cases (N = 36)
Sex			
- Female	95 (67.38%)	71 (67.62%)	24 (66.67%)
- Male	46 (32.62%)	34 (32.38%)	12 (33.33%)
Age			
- Mean ± SD (range)	48.26 ± 12.39 (18–74)	49.76 ± 11.57 (18–74)	43.89 ± 13.76 (18–72)
- Median	47	49	43.5
- <50 years	79 (56.03%)	54 (51.43%)	25 (69.44%)
- ≥50 years	62 (43.97%)	51 (48.57%)	11 (30.56%)
Ear side			
- Right	66 (46.81%)	47 (44.76%)	19 (52.78%)
- Left	75 (53.19%)	58 (55.24%)	17 (47.22%)
Surgical steps			
- Classic technique	16 (11.35%)	8 (7.62%)	8 (22.22%)
- Reversal technique	79 (56.03%)	56 (53.33%)	23 (63.89%)
- Modified classic technique	46 (32.62%)	41 (39.05%)	5 (13.89%)

SD: standard deviation.

**Table 2:** Preoperative and postoperative hearing data of patients treated by diode laser procedure.

Frequency (kHz)	Air Conduction Mean ± SD, Median (IQR)	Bone Conduction Mean ± SD, Median (IQR)	Air–Bone Gap Mean ± SD, Median (IQR)
Preoperative			
- 0.25 kHz	63.48 ± 13.37, 65 (15)	-	-
- 0.5 kHz	63.09 ± 12.06, 65 (10)	21.67 ± 9.57, 20 (15)	41.42 ± 9.85, 40 (10)
- 1 kHz	58.62 ± 13.04, 55 (15)	23.05 ± 10.15, 20 (15)	35.57 ± 10.13, 35 (10)
- 2 kHz	55.19 ± 16.27, 50 (20)	32.52 ± 14.78, 30 (20)	22.67 ± 11.47, 20 (15)
- 4 kHz	55.19 ± 18.07, 55 (25)	28.57 ± 14.90, 25 (25)	26.62 ± 12.58, 25 (15)
- 8 kHz	56.97 ± 20.88, 55 (21.5)	-	-
- PTA	58.02 ± 12.58, 56.25 (12.5)	26.45 ± 10.54, 25 (12.5)	31.57 ± 2.03, 31.25 (11.25)
Postoperative			
- 0.25 kHz	35.52 ± 12.23, 30 (15)	-	-
- 0.5 kHz	31.43 ± 12.68, 30 (15)	18.95 ± 8.73, 20 (10)	12.48 ± 9.41, 10 (10)
- 1 kHz	32.57 ± 13.43, 30 (20)	21.00 ± 10.82, 20 (15)	11.57 ± 8.59, 10 (10)
- 2 kHz	35.33 ± 16.48, 30 (25)	27.09 ± 14.59, 20 (20)	8.24 ± 7.66, 5 (5)
- 4 kHz	42.48 ± 20.58, 35 (30)	27.53 ± 17.21, 20 (25)	14.95 ± 10.23, 15 (15)
- 8 kHz	53.33 ± 22.31, 32.5 (35)	-	-
- PTA	35.45 ± 13.54, 50 (21.25)	23.64 ± 10.86, 20 (16.25)	11.81 ± 2.68, 8.75 (6.25)

SD: standard deviation; PTA: pure-tone average.

**Table 3:** Statistical comparison between preoperative and postoperative audiological results in both groups.

Study Group	Diode Laser Patients			Conventional Technique Patients		
Frequency (kHz)	Preoperative Mean ± SD, Median (IQR)	Postoperative Mean ± SD, Median (IQR)	*p*-Value *	Preoperative Mean ± SD, Median (IQR)	Postoperative Mean ± SD, Median (IQR)	*p*-Value *
Air conduction						
- 0.25 kHz	63.48 ± 13.37, 65 (15)	35.52 ± 12.23, 30 (15)	**<0.00001**	58.89 ± 13.89, 60 (20)	31.53 ± 13.46, 30 (20)	**<0.00001**
- 0.5 kHz	63.09 ± 12.06, 65 (10)	31.43 ± 12.68, 30 (15)	**<0.00001**	58.19 ± 11.03, 55 (15)	28.61 ± 12.85, 25 (15)	**<0.00001**
- 1 kHz	58.62 ± 13.04, 55 (15)	32.57 ± 13.43, 30 (20)	**<0.00001**	57.08 ± 13.65, 55 (11.25)	27.22 ± 12.62, 25 (10)	**<0.00001**
- 2 kHz	55.19 ± 16.27, 50 (20)	35.33 ± 16.48, 30 (25)	**<0.00001**	51.11 ± 16.13, 50 (20)	27.36 ± 13.65, 25 (15)	**<0.00001**
- 4 kHz	55.19 ± 18.07, 55 (25)	42.48 ± 20.58, 35 (30)	**<0.00001**	49.03 ± 16.42, 50 (15)	33.61 ± 16.33, 30 (25)	**<0.0001**
- 8 kHz	56.97 ± 20.88, 55 (21.5)	53.33 ± 22.31, 32.5 (35)	**0.0443**	52.50 ± 18.26, 55 (26.25)	45.97 ± 21.24, 42.5 (26.25)	0.161
- PTA	58.02 ± 12.58, 56.25 (12.5)	35.45 ± 13.54, 50 (21.25)	**<0.00001**	53.85 ± 12.73, 51.87 (12.5)	29.20 ± 12.06, 28.75 (13.75)	**<0.0001**
Bone conduction						
- 0.5 kHz	21.67 ± 9.57, 20 (15)	18.95 ± 8.73, 20 (10)	**0.003**	20.28 ± 10.28, 20 (10)	16.11 ± 11.28, 15 (10)	**0.034**
- 1 kHz	23.05 ± 10.15, 20 (15)	21.00 ± 10.82, 20 (15)	**0.005**	20.42 ± 11.36, 20 (11.25)	15.97 ± 11.39, 15 (10)	**0.036**
- 2 kHz	32.52 ± 14.78, 30 (20)	27.09 ± 14.59, 20 (20)	**<0.00001**	28.47 ± 13.35, 25 (17.5)	19.44 ± 13.35, 15 (15)	**0.002**
- 4 kHz	28.57 ± 14.90, 25 (25)	27.53 ± 17.21, 20 (25)	0.063	26.39 ± 13.71, 20 (16.25)	23.19 ± 14.35, 15 (16.25)	0.171
- PTA	26.45 ± 10.54, 25 (12.5)	23.64 ± 10.86, 20 (16.25)	**0.001**	23.89 ± 11.14, 20 (12.81)	18.68 ± 11.12, 16.87 (11.5)	0.332
Air–bone gap						
- 0.5 kHz	41.42 ± 9.85, 40 (10)	12.48 ± 9.41, 10 (10)	**<0.00001**	37.91 ± 8.73, 35 (15)	12.50 ± 6.27, 10 (5)	**<0.00001**
- 1 kHz	35.57 ± 10.13, 35 (10)	11.57 ± 8.59, 10 (10)	**<0.00001**	36.66 ± 10.28, 35 (11.25)	11.25 ± 5.26, 10 (5)	**<0.00001**
- 2 kHz	22.67 ± 11.47, 20 (15)	8.24 ± 7.66, 5 (5)	**<0.00001**	22.64 ± 10.92, 20 (11.25)	7.92 ± 3.46, 7.5 (5)	**<0.00001**
- 4 kHz	26.62 ± 12.58, 25 (15)	14.95 ± 10.23, 15 (15)	**<0.00001**	22.64 ± 10.03, 20 (15)	10.42 ± 5.26, 10 (10)	**<0.00001**

SD: standard deviation; PTA: pure-tone average. * Mann–Whitney test, *p*-value < 0.05.

**Table 4:** Statistical analysis for subgroups.

Subgroups	Postop. AC PTA	*p*-Value *	Postop. BC PTA	*p*-Value *	ABG 500 Hz	*p*-Value *	ABG 1000 Hz	*p*-Value *	ABG 2000 Hz	*p*-Value *	ABG 4000 Hz	*p*-Value *
Gender												
- Female	32.71		21.91		12.47		10.74		7.32		12.68	
- Male	36.22	0.290	23.34	0.920	12.50	0.627	13.04	0.139	9.89	0.057	16.09	0.131
Age												
- <50 years old	31.30		20.51		11.89		10.89		7.85		12.53	
- ≥50 years old	37.12	0.980	24.76	0.681	13.23	0.804	12.26	0.640	8.55	**0.018**	15.40	0.248
Surgical steps												
- Classic technique	34.45		25.54		9.69		8.75		5.63		11.56	
- Reversal technique	32.94		21.25		12.85		12.28		8.55		13.10	
- Modified classic technique	35.22	0.665	23.21	0.154	12.82	0.223	11.09	0.163	8.37	0.261	15.76	0.593

AC: air conduction; BC: bone conduction; PTA: pure-tone average; ABG: air–bone gap. * linear regression model, *p*-value < 0.05.

## Data Availability

Data are available upon request due to privacy and ethical reasons.

## Abbreviations

The following abbreviations are used in this manuscript:

CHL	Conductive hearing loss
SNHL	Sensorineural hearing loss
KTP	Potassium-Titanyl-Phosphate
ABG	Air–bone gap
EAC	External auditory canal
PTA	Pure-tone average
AC	Air conduction
BC	Bone conduction
IQR	Interquartile range (Q_3_–Q_1_)
